# The Impact of Lateral Gene Transfer in *Chlamydia*

**DOI:** 10.3389/fcimb.2022.861899

**Published:** 2022-03-07

**Authors:** Hanna Marti, Robert J. Suchland, Daniel D. Rockey

**Affiliations:** ^1^Institute of Veterinary Pathology, Vetsuisse-Faculty, University of Zurich, Zurich, Switzerland; ^2^Division of Allergy and Infectious Diseases, Department of Medicine, University of Washington, Seattle, WA, United States; ^3^Department of Biomedical Sciences, Carlson College of Veterinary Medicine, Oregon State University, Corvallis, OR, United States

**Keywords:** horizontal gene transfer, homologous recombination, *Chlamydiaceae*, RecBCD, RecFOR, co-infection, membrane proteins, DNA uptake

## Abstract

Lateral gene transfer (LGT) facilitates many processes in bacterial ecology and pathogenesis, especially regarding pathogen evolution and the spread of antibiotic resistance across species. The obligate intracellular chlamydiae, which cause a range of diseases in humans and animals, were historically thought to be highly deficient in this process. However, research over the past few decades has demonstrated that this was not the case. The first reports of homologous recombination in the *Chlamydiaceae* family were published in the early 1990s. Later, the advent of whole-genome sequencing uncovered clear evidence for LGT in the evolution of the *Chlamydiaceae*, although the acquisition of tetracycline resistance in *Chlamydia (C.) suis* is the only recent instance of interphylum LGT. In contrast, genome and *in vitro* studies have shown that intraspecies DNA exchange occurs frequently and can even cross species barriers between closely related chlamydiae, such as between *C. trachomatis*, *C. muridarum*, and *C. suis*. Additionally, whole-genome analysis led to the identification of various DNA repair and recombination systems in *C. trachomatis*, but the exact machinery of DNA uptake and homologous recombination in the chlamydiae has yet to be fully elucidated. Here, we reviewed the current state of knowledge concerning LGT in *Chlamydia* by focusing on the effect of homologous recombination on the chlamydial genome, the recombination machinery, and its potential as a genetic tool for *Chlamydia.*

## Introduction

The gram-negative *Chlamydiaceae* family consists of several pathogenic species that cause diseases ranging from pneumonia to sexually transmitted infections (STI) in humans, livestock, pets, and wildlife. In humans, *Chlamydia (C.) trachomatis* is the cause of chronic eye infections leading to blindness (trachoma), and STI, while *C. pneumoniae* induces community-acquired pneumonia. *C. psittaci* is a zoonotic pathogen primarily detected in birds causing flu-like symptoms to life-threatening pneumonia in humans. *C. abortus* is the cause of ovine enzootic abortion (OEA) in sheep and goats and may also induce miscarriage in women. In contrast, *C. suis*, another chlamydial species with zoonotic potential, is found in the eyes and intestinal tract of pigs, often remaining asymptomatic (Dean et al., 2013; De Puysseleyr et al., 2014; De Puysseleyr et al., 2017; Sachse and Borel, 2020; Jordan et al., 2020). Although the chlamydial obligate intracellular life cycle is reflected by extensive streamlining and reduction of the genome (Palmer, 2002; Toft and Andersson, 2010), the *Chlamydiaceae* possess a number of genes involved in DNA uptake, recombination, and repair (Stephens et al., 1998; LaBrie et al., 2019) enabling intra- and interspecies lateral gene transfer (Suchland et al., 2009; Somboonna et al., 2011; Joseph and Read, 2012; Joseph et al., 2015; Marti et al., 2017).

Lateral, or horizontal, gene transfer (LGT) involves transfer of genetic material (DNA) from one cell to another and subsequent integration into the genome of the recipient cell. In bacteria, DNA transfer is primarily facilitated by transduction (bacteriophage infection), conjugation/mobilization/conduction (plasmid transfer), and transformation (uptake of naked DNA). DNA integration is then directed by homologous or non-homologous recombination (Redfield, 2001).

Here, we will review the current state of knowledge regarding lateral gene transfer (LGT) in the *Chlamydiaceae* by focusing on i) the impact of recombination on the *Chlamydiaceae* genome, ii) the homologous recombination machinery of the *Chlamydiaceae*, and iii) homologous recombination as a potential genetic tool.

## The Impact of Homologous Recombination on the Chlamydial Genome

The first reports providing evidence for intrastrain recombination within *C. trachomatis* were published in the 1990s and were based on gene-specific sequence analysis of *ompA*, which encodes the major outer membrane protein (MOMP) (Lampe et al., 1993; Brunham et al., 1994; Hayes et al., 1994; Hayes et al., 1995). Whole-genome analysis of laboratory and clinical strains later revealed that recombination events occurred across the entire genome during the evolution of *C. trachomatis* (Jeffrey et al., 2010; Joseph et al., 2011; Harris et al., 2012), as well as other chlamydial species such as *C. pneumoniae*, *C. psittaci*, and *C. suis* (Read et al., 2013; Roulis et al., 2015; Joseph et al., 2016; Seth-Smith et al., 2017b). Interestingly, investigation of *C. abortus* revealed no sign of recombination in currently circulating strains (Joseph et al., 2015; Seth-Smith et al., 2017a).

Whole-genome analyses further identified regions of high genomic diversity and, in parallel, regions with apparently higher rates of recombination. In *C. trachomatis* and *C. pneumoniae*; these included *ompA* (Hayes et al., 1994), the polymorphic membrane protein-encoding genes (*pmps*) (Jordan et al., 2001; Rocha et al., 2002; Brunelle and Sensabaugh, 2006), *incA*, and the translocated actin-recruiting phosphoprotein-encoding gene *tarp* (Joseph et al., 2012; Joseph and Read, 2012; Roulis et al., 2015), as well as the plasticity zone (PZ) (Jeffrey et al., 2010). Tarp is an important effector protein involved in the re-structuring of the host cytoskeleton (Tolchard et al., 2018). The PZ encodes for a range of different genes that are hypothesized to have important functions in the pathogenicity of the chlamydiae and may be a site of increased susceptibility for DNA uptake, genetic variation, and functional gene loss (Read et al., 2000; Thomson et al., 2008; Rajaram et al., 2015).

In the evolution and diversification of the *Chlamydiaceae* family, widespread gene rearrangement and translocation were identified between *C. pneumoniae* and *C. trachomatis* (Tillier and Collins, 2000). Furthermore, LGT events were detected both within and among the four major strain clusters of *C. trachomatis*, namely, the lymphogranuloma venereum (LGV), the trachoma, and two urogenital (T1, T2) clusters (Hadfield et al., 2017). Some studies have shown that this could have a clinical impact in terms of virulence and epidemiology (Somboonna et al., 2011; Andersson et al., 2016; Hadfield et al., 2017; Borges et al., 2019). Moreover, the effect of recombination can vary greatly between the four above-mentioned lineages of *C. trachomatis*, with the ocular strains being less affected than the urogenital lineages and the clonal LGV lineage having undergone no significant re-combination (Hadfield et al., 2017; Seth-Smith et al., 2021).

Overall, current data suggest that *C. psittaci*, *C. pneumoniae*, and *C. suis* have undergone higher rates of recombination than the entirety of the four *C. trachomatis* lineages. However, direct comparison between studies remains difficult due to the varying number of available genomes per species and because of the different approaches used to calculate *r/m* and other statistics that aim to quantify the recombination rate of a population (Read et al., 2013; Joseph et al., 2015; Roulis et al., 2015; Joseph et al., 2016; Hadfield et al., 2017; Seth-Smith et al., 2017b).

Additionally, one study proposed that ribosomal binding sites and tRNA may be associated with recombinant breakpoints (Gomes et al., 2006). However, these findings have yet to be confirmed by *in vitro* studies. So far, *in vitro* studies dealing with LGT following co-infection found little evidence for specific patterns, regions, or sites of recombination (Jeffrey et al., 2013; Marti et al., 2021), although there are notable differences between interspecies and intraspecies crosses, with intraspecies crosses generally leading to a higher proportion of donor DNA in the recombinant strains (Suchland et al., 2019). Moreover, the same study found that the replication termination is a target for interspecies recombination.

One very interesting chlamydial species in the context of LGT is *C. suis.* It is the only chlamydial species to have naturally obtained a resistance gene, *tetA*(C), which encodes a tetracycline efflux pump. This resistance allele and its genetic content were integrated as a genomic island (Tet-island) into the *C. suis* chromosome in an invasion-like gene (*inv*), probably during a transposition event directed by the transposase-encoding insertion sequence IScs605, although the exact mode of transmission and integration could not be replicated in an *in vitro* model involving *C. suis* (Dugan et al., 2004; Dugan et al., 2007). This Tet-island is the only evidence for recent acquisition of foreign DNA from other bacteria in *Chlamydia* spp. It shares high nucleotide identity with a pRAS3-type plasmid from the fish pathogen *Aeromonas salmonicida ssp. salmonicida* (Massicotte et al., 2019). It has been hypothesized that the plasmid was transferred *via* feeding of pigs with fish meal (Sandoz and Rockey, 2010) and was selected for with the use of tetracycline as a growth promoter in pig production facilities (Dugan et al., 2004). The use of tetracycline in pigs as prophylactic and therapeutic treatment has been shown to increase the rate of *C. suis* strains positive for *tetA*(C) (Borel et al., 2012; Borel et al., 2016; Wanninger et al., 2016), and whole-genome analysis and *in vitro* studies have indicated that intraspecies spread of *tetA*(C) is the result of homologous recombination (Joseph et al., 2016; Marti et al., 2017; Marti et al., 2021).

It is concerning that the *tetA*(C) marker can readily and stably integrate into *C. trachomatis* and *C. muridarum* strains *in vitro* (Suchland et al., 2009), leading to the possibility that these strains could acquire tetracycline resistance in clinical settings. This possibility was strengthened when *C. suis* was detected and isolated from the eyes, feces, and pharynges of veterinarians, pig farmers, and abattoir workers, although tetracycline-resistant *C. suis* strains have yet to be isolated from human samples (Dean et al., 2013; De Puysseleyr et al., 2014; De Puysseleyr et al., 2017). Because ocular *C. trachomatis* infection (inclusion conjunctivitis) through autoinoculation with genital *C. trachomatis* strains D-K has been reported (Haller-Schober and El-Shabrawi, 2002), the possibility of Tet-island transmission from *C. suis* to *C. trachomatis* cannot be excluded.

One remarkable finding that has emerged during natural LGT in *Chlamydia* is the difference between cross-species vs. intraspecies genetic transfer occurrence. We envision a model where LGT within the inclusion is very common, to the extent that clonal *C. trachomatis* recombines regularly in inclusions that form following infection with a single EB. The selective driver for such common genetic exchange is currently unclear but would be consistent with the principles of Muller’s Ratchet, where it is hypothesized that random mutation in haploid organisms would lead to fully degraded genomes in the absence of LGT (Joseph et al., 2011; Takeuchi et al., 2014). Other selective drivers, such as the Hill–Robertson effect, where the overall responsiveness to selection is reduced in finite populations, may also play a role (Joseph et al., 2011). Therefore, we propose that the reason for such common intraspecies LGT is to regenerate or maintain wild-type genomes in an intracellular environment that otherwise might be considered stressful and mutagenic (MacLean et al., 2013; Maharjan and Ferenci, 2017).

## The Chlamydial Recombination Machinery

**Figure 1:**
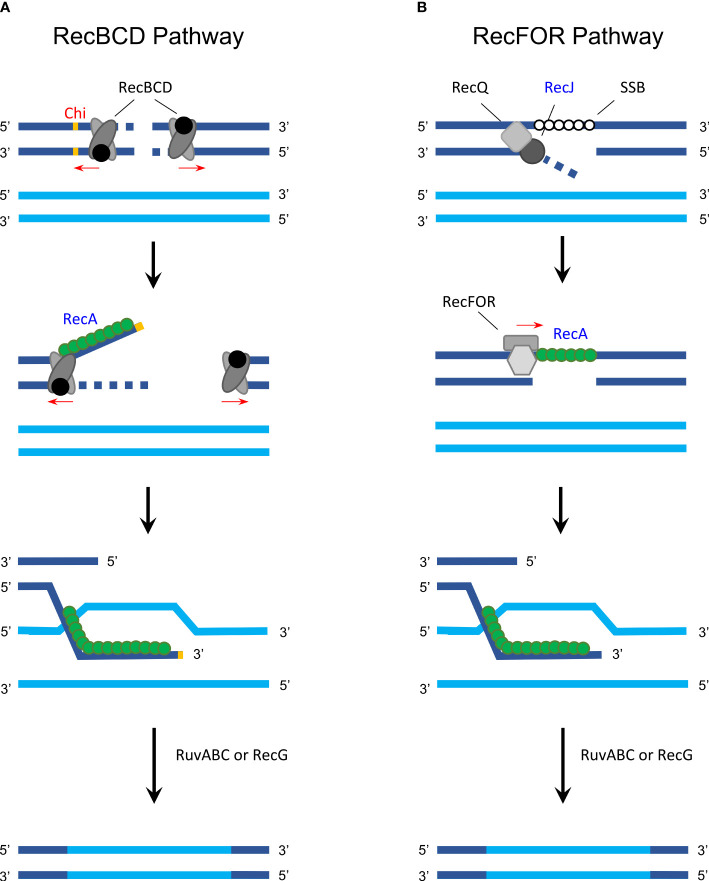
Homologous recombination in gram-negative bacteria. **(A)** The RecBCD pathway is activated following a double-strand break that causes the RecBCD complex to bind on both ends and degrade DNA from the 3′ to 5′ end until one of the complexes encounters a Chi site. RecBCD then degrades the DNA from to 5′ to 3′ end while RecA (green) can bind to the 3′ extension. Next, the RecA-covered single-strand DNA invades a homologous sequence (synapsis formation) and RuvABC (with or without RecG) is used to resolve the Holliday junction, exchanging DNA *via* recombination. **(B)** In the RecFOR pathway, a single-strand break is first unwound with helicase RecQ and degraded with RecJ, while single-stranded binding protein (SSB) attaches to the exposed strand. This is followed by RecFOR promoting the replacement of SSB with RecA followed by the same process as described in the RecBCD pathway. Proteins that were analyzed in detail regarding its function and activity in *Chlamydia* are labeled in blue; protein/sites that are unknown or inexistent in *Chlamydia* are labeled in red. The figure was modified from Rocha et al. (2005), Figure 1, and Snyder et al. (2013), Figures 10.2, 10.3, and 10.4.

Homologous recombination allows inter- and intragenomic exchange of DNA and therefore plays a crucial role in genetic diversification and DNA repair (Rocha et al., 2005). In bacteria, homologous recombination consists of two major pathways, the RecBCD (**Figure 1A**) and the RecFOR pathway (**Figure 1B**), both of which facilitate DNA exchange between a com-plementary sequence and single-strand DNA (ssDNA) using the RecA protein (Rocha et al., 2005).

In *Chlamydia*, whole-genome sequencing revealed that the genome contains various genes from the recombination and DNA repair machinery (Stephens et al., 1998; Azuma et al., 2006). However, only few studies have investigated the function and exact mechanism of the chlamydial homologous re-combination machinery. The first chlamydial recombination-associated protein to be analyzed was RecA in *C. trachomatis*, which was found to have moderate recombinational activity and possessed low efficiency after DNA damage by UV radiation compared to other bacteria (Hintz et al., 1995; Zhang et al., 1995). Chlamydial RecJ has a similar function as that of other gram-negative bacteria, namely, exonuclease activity in RecBCD-independent and conjugational recombination (Hsia and Bavoil, 1996; Rocha et al., 2005). It is expected that the RecBCD and RecFOR pathways of *Chlamydia* work similarly to that of *E. coli*, including formation and resolution of Holliday junctions due to the presence of *ruv* genes (Bastidas and Valdivia, 2016).

Some *Chlamydia*-specific particularities and open questions remain. For example, the histone-like protein Hc1 is involved in the condensation of the chlamydial nucleoid and inhibits RecA activity. Interestingly, however, Hc1 only inhibits its repair and not its recombinational activity (Ennis et al., 2000). For the RecBCD pathway, the exact identity of *Chlamydia*-specific Chi sites is unknown, as indicated in **Figure 1** (Gomes et al., 2006).

Overall, it appears that the recombination machinery of the *Chlamydiaceae* family is complete, which underlines the importance of homologous recombination for a bacterial species that has undergone significant gene reduction (Palmer, 2002). However, more studies are necessary to confirm current assumptions that are only based on genomic data. With increasing options to genetically modify the chlamydiae (Valdivia and Bastidas, 2018), these investigations have become a possibility. First advances have already been made in recent years by the creation of knockout mutants in which genes involved in LGT are inactivated (Kokes et al., 2015; LaBrie et al., 2019; Wang et al., 2019). Currently available knockout mutants concerning LGT involving DNA uptake and ho-mologous recombination are listed in **Table 1**.

**Table 1:** List of knockout mutants concerning genes involved in lateral gene transfer.

Strain name	Species/strain	Mutation	Locus (gene), function	Literature
UWCM026	*Cm/*Nigg	Transposon mutant (knockout)	TC0212 (*rmuC*), DNA recombination protein	Wang et al., 2019
UWCM031	*Cm/*Nigg	Transposon mutant (knockout)	TC0302 (*recD*), RecBCD complex	Wang et al., 2019
ctl10707 (ct447)	*Ct*/L2	Transposon mutant (knockout)	CT447 (*recJ*), RecFOR pathway	LaBrie et al., 2019
ctl10730 (ct470)	*Ct*/L2	Transposon mutant (knockout)	CT470 (*recO*), RecFOR pathway	LaBrie et al., 2019
CTL2M934	*Ct*/L2	Transposon mutant, nonsense SNV *^a^* (knockout)	CT339 (*com*EC), DNA uptake (transformation)	Kokes et al., 2015; LaBrie et al., 2019
CTL2M_Pool 27	*Ct*/L2	Nonsense SNV *^a^* (knockout)	CT298 (*radA*), DNA repair protein (recombinase)	Kokes et al., 2015
CTL2M_Pool 23	*Ct*/L2	Nonsense SNV *^a^* (knockout)	CT040 (*ruvB*), Holliday junction ATP-dependent DNA helicase	Kokes et al., 2015
CTL2M_Pool 30	*Ct*/L2	Nonsense SNV *^a^* (knockout)	CT825 (*rmuC*), DNA recombination	Kokes et al., 2015
CTL2M924	*Ct*/L2	Nonsense SNV *^a^* (knockout)	CT660 (*gyrA2*), DNA gyrase subunit 2, DNA replication	Kokes et al., 2015

Cm, C. muridarum; Ct, C. trachomatis.

a Single-nucleotide variant (SNV) created with chemical mutagenesis. Nonsense mutants were listed in Kokes et al. (2015).

## Homologous Recombination and Genetic Engineering

Genetic manipulation is an indispensable tool to understanding the biology of eukaryotic and prokaryotic cells. In the *Chlamydia* research field, tools for genetic modification have only recently been developed. The currently available methods have been reviewed in detail (Bastidas and Valdivia, 2016); therefore, we will only discuss genetic engineering in the context of homologous recombination.

The first report of successful, albeit transient, transformation of *Chlamydia* was published in the 1990s (Tam et al., 1994). Fifteen years later, a study could stably introduce kasugamycin and spectinomycin resistance into *C. psittaci* by introducing a pUC derivative, which carried the ribosomal RNA (rrn) region of *C. psittaci* with resistance-inducing point mutations, into the wild type using electroporation (Binet and Maurelli, 2009). Shuttle vectors comprising an *E. coli* vector and the chlamydial plasmid later allowed stable and reproducible transformation of *Chlamydia* (Wang et al., 2011), overhauling the field of *Chlamydia* genetics.

One of the remaining challenges of genetic manipulation of *Chlamydia* spp. is the inability of the pathogen to maintain plasmids with replication systems that do not include the native chlamydial plasmid. There has been significant progress in this field when a very recent report used a recombinant construct based on a broad-spectrum plasmid from *Bordetella pertussis* (pBBR1 MCS4) to transform a *C. trachomatis* L2 strain. This plasmid, pBVR1, contained *C. trachomatis* genomic sequences that allowed integration of the element into the *C. trachomatis* chromosome. This construct was maintained as both an episome and an integrated element in transformed strains. It is expected that further work with the pBBR1 vector system will perhaps allow the maintaining of non-chlamydial-plasmid-based genetic elements in transformed strains (Garvin et al., 2021).

Additionally, co-culture models were established as an alternative method to genetically modify the chlamydiae by co-infecting cells with two *C. trachomatis* strains, each carrying resistance-conferring mutations to either ofloxacin, lincomycin, trimethoprim, or rifampicin, and selecting for double-resistant recombinants. These studies detected recombination frequencies of 10^-4^ to 10^-3^ and further proposed that LGT likely played an important part in chlamydial evolution (DeMars et al., 2007; DeMars and Weinfurter, 2008). Similar protocols further demonstrated that interspecies transfer of the Tet-island from *C. suis* to *C. trachomatis* and *C. muridarum*, but not the more distantly related *C. caviae*, is possible. While *C. muridarum* obtained an approximately 100 kb-long sequence (the Tet-island and surrounding genes) as the result of a homologous recombination-mediated crossover event, co-infection of *C. suis* and *C. trachomatis* produced a mosaic strain with three instead of two *rrn* operons (Suchland et al., 2009). Interspecies transfer of the Tet-island *via* homologous recombination has been shown for *C. trachomatis* (Jeffrey et al., 2013) and *C. suis*, both in the presence (Marti et al., 2021) and absence (Marti et al., 2017) of double selection. Interestingly, comparison of *in vitro*-generated recombinant strains with clinical strains demonstrated that there are statistically more breakpoints in *in vitro C. trachomatis* strains compared to clinical strains, especially in the resistance-conferring genes *rpoB* (rifamycin group) and *gyrA* (ofloxacin) (Srinivasan et al., 2012).

The principle of co-infection and selection for recombinants has since become crucial in genetic engineering of the *Chlamydia*. For example, it has been used as a mapping tool in forward genetics, either by chemical mutagenesis and subsequent selection of recombinants using resistance markers (Nguyen and Valdivia, 2013; Nguyen and Valdivia, 2014) or by employing markerless recombination approaches (Brothwell et al., 2016). Moreover, suicide vectors that allow gene deletion following homologous recombination have been successfully constructed and used (Mueller et al., 2016; McKuen et al., 2017; Mueller et al., 2017). Finally, the principle of interspecies LGT has been exploited to create a hybrid strain library of *C. trachomatis*/*C. muridarum* crosses: a *tet-*resistant *C. trachomatis* strain was crossed *in vitro* with *C. muridarum* strains mutated by the plasmid-based Himar transposition system that randomly integrated a chloramphenicol marker into the genome (Suchland et al., 2019; Wang et al., 2019). This method was then used to produce PZ chimeras where the *C. muridarum* PZ replaced that of *C. trachomatis*, which demonstrated that the *C. muridarum*-specific large putative cytotoxins are not responsible for cytopathic and cytotoxic effects. This switch-out method further led to the detection of an inclusion protein, CT147, and CTL0402, which plays a role in the inclusion integrity (Dimond et al., 2021). A back-crossing strategy, which is a technique that can be used to effect functional complementation of mutants, was then used to restore both the wild-type genotype and phenotype.

## Discussion and Outlook

Research over the past two decades has identified a paradox with regard to the genetic exchange and transformation within chlamydiae. First, decades of effort have demonstrated that *Chlamydia* spp. are very challenging to transform genetically, and even now the use of genetic systems remains difficult. This challenge is further exemplified by the near absence of LGT by members of the chlamydiae from bacteria across species. In contrast, some chlamydiae, notably *C. trachomatis*, undergo regular intraspecies LGT between different isolates.

The rarity of interphylum LGT events is contrasted with the presence of abundant LGT machinery retained in the chlamydial genome, even despite the considerable gene reduction following adaptation to its intracellular life cycle (Toft and Andersson, 2010). As described in this review, the recombination machinery of *Chlamydia* is complete, although some questions remain, and of the three major known forms of DNA uptake, two have been described. Specifically, while the *Chlamydiaceae* family does not possess a known conjugation machinery (Greub et al., 2004), transformation and transduction are possible. For example, one recent study showed that CT336 in *C. trachomatis*, a protein with limited sequence similarity to the *Bacillus* ComEC protein, plays an important role in DNA uptake *via* transformation (LaBrie et al., 2019). However, the same study noted that other important genes involved in the uptake of free dsDNA, namely, homologs of PilQ, ComEA, and DprA, are absent, which led to the conclusion that natural transformation in the *Chlamydiaceae* is different from that of other gram-negative bacteria, similar to *Helicobacter pylori* (LaBrie et al., 2019). Furthermore, chlamydiaphages (chlamydial bacteriophages) in the *Chla-mydiamicrovirus* genus have been described in various chlamydial species such as *C. psittaci*, *C. abortus*, *C. felis*, *C. caviae*, *C. pecorum*, and *C. pneumoniae*, but not in the more distantly related *C. suis*, *C. muridarum*, and *C. trachomatis* (Pawlikowska-Warych et al., 2015; Bastidas and Valdivia, 2016). Effort to use these phages to facilitate genetic in-troduction has not yet been successful, but perhaps future research will identify ways to use transduction as a tool of genetic exchange in *Chlamydia*.

In conclusion, despite significant progress in our un-derstanding of LGT in *Chlamydia*, many open questions remain. For example, most *in vitro* studies concerning LGT and homologous recombination have been conducted with *C. suis*, *C. muridarum*, and *C. trachomatis.* While it is possible to induce competence in *C. psittaci*, *C. felis*, and *C. pneumoniae* (Shima et al., 2018; Shima et al., 2020), we know very little about their DNA uptake system and if it is similar to that of *C. trachomatis*, *C. muridarum*, and *C. suis.* There may be a different mechanism for these species, as there appears to be a barrier of recombination between *C. suis*, *C. trachomatis*, and *C. caviae* (Suchland et al., 2009). Equipped with new genetic tools and a more extensive knowledge of LGT in the *Chlamydiaceae* family, we can tackle these challenging questions and further explore the biology of these complex bacteria.

## Author Contributions

HM, RS, and DR substantially contributed to the conception and design of the manuscript and reviewed the literature. All authors drafted and/or critically revised the manuscript, finally approved the version to be published, and agreed to be accountable for all aspects of the work.

## Funding

This work was funded in part by the National MD-Ph.D. scholarship program organized by the Swiss Academy of Medical Sciences (SAMW), sponsored by the Swiss National Science Foundation (SNSF; Grant No. 323530_177579, awarded to HM from September 2017 to August 2020).

## Conflict of Interest

The authors declare that the research was conducted in the absence of any commercial or financial relationships that could be construed as a potential conflict of interest.

## Publisher’s Note

All claims expressed in this article are solely those of the authors and do not necessarily represent those of their affiliated organizations, or those of the publisher, the editors and the reviewers. Any product that may be evaluated in this article, or claim that may be made by its manufacturer, is not guaranteed or endorsed by the publisher.
